# Entropy-Based Analysis of Emotional Dynamics in Doctor–Patient Co-Decision in Primary Care

**DOI:** 10.3390/e28101046

**Published:** 2026-09-23

**Authors:** Ayrton Sarango, Juan Aguarón, Rodrigo Aznar, Daniel Bordonaba, Cristina Moreno-Loscertales, José María Moreno-Jiménez, Jorge Navarro

**Affiliations:** 1 Escuela Técnica Superior de Ingenieros Informáticos, Universidad Politécnica de Madrid, 28040 Madrid, Spain; ayrton.sarango@alumnos.upm.es; 2 Grupo Decisión Multicriterio Zaragoza, Facultad de Economía y Empresa, Universidad de Zaragoza, 50009 Zaragoza, Spain; aguaron@unizar.es (J.A.); dbordona@unizar.es (D.B.); moreno@unizar.es (J.M.M.-J.); 3 Hospital San Juan de Dios, 50006 Zaragoza, Spain; rodrigo.aznar@sjd.es; 4 Servicio de Apoyo Metodológico, Estadístico y Documental (SAMEyD), Instituto Aragonés de Ciencias de la Salud (IACS), 50009 Zaragoza, Spain; dbordonaba.iacs@aragon.es; 5 Grupo de Investigación en Neurociencias, Instituto de Investigación Sanitario Aragón, 50009 Zaragoza, Spain; cmoreno@salud.aragon.es

**Keywords:** entropy, primary care, doctor–patient interaction, shared decision making, natural language processing, sentiment analysis, emotional dynamics, hypertension, dyslipidemia

## Abstract

Decision-making in healthcare involves both clinical evidence and emotional and cognitive processes that are often difficult to formalize. While sentiment analysis has been widely used to capture emotional information in clinical interactions, it does not fully account for how emotional categories are distributed across interactions. This study proposes an entropy-based approach to complement sentiment analysis by quantifying the diversity and distribution of emotional states expressed during doctor–patient consultations. A dataset of 30 primary care consultations involving patients with hypertension (HTA) and dyslipidemia (DLM) was analyzed using natural language processing techniques. Emotional information was extracted using the NRC Emotion Lexicon, and normalized Shannon entropy was computed at the intervention level. Intervention-based, role-based, and phase-based analyses were conducted. The results show that emotional entropy varies throughout the consultation and across consultation phases. Consultation-level analyses showed higher overall entropy in HTA than in DLM consultations, while no significant overall effect of the speaker role was found. Entropy also varied significantly in consultation phases, although the disease-by-phase interaction was not significant. Robustness analyses supported the stability of these patterns, and the negligible association with sentiment valence indicates complementary information. These findings highlight entropy as a complementary measure for analyzing emotional dynamics in clinical interactions.

## 1. Introduction

Decision-making in real-world contexts must incorporate both objective information associated with data and subjective information associated with human preferences and values [1]. In practice, it emerges from the interaction between structured knowledge, personal experience, and affective factors that are often difficult to formalize. This is especially evident in healthcare, where decisions are not only guided by clinical evidence but also shaped by patients’ perceptions, emotions, and preferences, as well as by Healthcare Cognitive Decision Support Systems (HC-DSS) [2,3].

In recent years, sentiment analysis has been increasingly used to capture emotional information embedded in textual data [4,5,6,7]. While these approaches provide useful insights into the polarity and type of emotions expressed, they do not fully capture how emotional categories are distributed across an interaction.

From an information-theoretic perspective, entropy offers a natural way to address this limitation [8,9,10,11]. By measuring the distribution of emotional states, entropy allows us to characterize the distribution and diversity of emotional categories within an interaction. This perspective provides a quantitative method for characterizing emotional distributions without assuming a direct correspondence with underlying psychological states [12].

Entropy-based approaches have been applied to the analysis of human behavior and communication processes, providing a quantitative framework to analyze emotional dynamics and the distribution of emotional categories, including differences across roles and consultation phases. Entropy has been used to study linguistic structure and information processing in natural language and for studying emotional and linguistic patterns in textual data [8,9,13,14]. These approaches enable the formalization of human communication as probabilistic systems, where emotional and linguistic information is distributed across multiple states and evolves dynamically along the interaction.

Beyond language analysis, entropy has also been incorporated into models of human decision-making as a quantitative framework for representing uncertainty in probabilistic systems [12]. From this perspective, emotional expressions can be understood as probabilistic distributions over affective states, making entropy a natural tool to characterize how emotional categories are distributed within an interaction. In this study, entropy is interpreted in an information-theoretic sense and should not be understood as a direct measure of psychological uncertainty. This distinction is particularly important in healthcare interactions, where information-theoretic measures should not be assumed to represent underlying psychological states without independent validation.

However, despite these advances, the application of entropy-based measures to doctor–patient interactions and shared decision-making processes remains limited. Existing approaches in healthcare have primarily focused on sentiment polarity or discrete emotional categories, without explicitly addressing the structural properties underlying emotional dynamics and this gap is particularly relevant in clinical contexts, where information exchange is accompanied by emotional expressions that may vary throughout the interaction. In this context, the present study extends entropy-based analysis to primary care consultations, providing a quantitative framework to characterize emotional dynamics and entropy patterns in doctor–patient co-decision processes. The objective of this study is exploratory and focuses on assessing whether emotional entropy can provide complementary information to conventional sentiment analysis in doctor–patient interactions.

In this framework, entropy is interpreted as a structural property of the distribution of emotional states, capturing the degree of concentration or dispersion of emotional categories within the interaction.

Primary care consultations provide a suitable setting to explore these issues. They represent complex, dynamic interactions in which clinical information, patient preferences, and emotional responses evolve simultaneously. In such settings, doctor–patient co-decision (commonly framed as Shared Decision Making—SDM) depends not only on the information exchanged, but also on how that information is perceived and emotionally processed by the patient [15,16].

Two chronic conditions frequently managed in primary care—hypertension (HTA) and dyslipidemia (DLM)—were selected as case studies due to their distinct clinical and experiential characteristics. While both are highly prevalent cardiovascular risk factors [17,18], they differ in how patients perceive and experience them. Hypertension is often associated with symptoms, perceived immediacy of risk, and greater psychological burden, whereas dyslipidemia is typically asymptomatic and primarily linked to long-term risk.

From an emotional and informational perspective, these differences make HTA and DLM suitable for exploring whether the diversity and distribution of detected emotional categories differ across clinical contexts.

Despite the growing interest in incorporating affective information into HC-DSS, existing approaches remain largely focused on sentiment polarity or discrete emotions [1,19], overlooking the structural properties of emotional dynamics. Little attention has been paid to the role of entropy as a measure of emotional distributional structure in doctor–patient interactions.

Previous work has explored sentiment-aware decision support systems in healthcare contexts, highlighting the relevance of emotional dynamics in doctor–patient interactions [1]. However, these approaches do not explicitly account for the distributional complexity of emotional categories.

This study proposes an entropy-based approach to analyze emotional dynamics in primary care consultations. Using text transcriptions from real doctor–patient interactions, we examine how emotional entropy evolves during the consultation and across different clinical conditions, thereby providing a complementary perspective to traditional sentiment analysis and offering a quantitative view of emotional dynamics in co-decision processes. These interactions were collected within the MESCAPE project (Mejora Extrahospitalaria de la Salud en los Centros de Atención Primaria y Emociones), a multidisciplinary initiative focused on analyzing emotional and decisional processes in primary care settings, which provides a suitable context for studying emotional dynamics in real consultations.

## 2. Materials and Methods

The methodological framework of this study integrates sentiment analysis and entropy-based measures to examine emotional dynamics in doctor–patient consultations. The approach combines natural language processing techniques with information-theoretic metrics to assess both the mood and the distributional structure of emotional expressions.

The analysis is based on real primary care consultations involving patients diagnosed with hypertension (HTA) and dyslipidemia (DLM). Emotional information is extracted from textual data and subsequently processed through a structured pipeline including data collection, pre-processing, sentiment analysis, and entropy-based modelling. Figure 1 illustrates the methodological pipeline used in the study. The process consists of five stages: data collection, data preprocessing, sentiment analysis, entropy-based analysis, and comparative analysis. This workflow summarizes how emotional information is extracted from consultation transcripts and subsequently analyzed to characterize emotional dynamics throughout the consultation process.

### 2.1. Data Collection

A dataset of primary care consultations was constructed; it included patients diagnosed with hypertension (HTA) and dyslipidemia (DLM). A total of 30 doctor–patient consultations (15 per condition) were audio-recorded in primary care centers. All participants provided informed consent in accordance with the ethical approval granted by the Ethics Committee of Aragón, Spain (CEICA).

The recordings were transcribed into text using WhisperX 3.1.1. Speaker identification and segmentation were performed with PyAnnote 2.1.1, allowing each intervention to be attributed to either the doctor or the patient. The resulting dataset consists of structured textual interactions ready for sentiment and entropy-based analysis.

### 2.2. Data Preprocessing

The transcribed texts were manually reviewed to correct transcription errors and ensure consistency in formatting. Each intervention was verified to guarantee correct attribution to the corresponding speaker. Text normalization procedures were applied to remove noise and standardize linguistic structures, facilitating subsequent analysis.

This preprocessing step ensures the reliability of the extracted emotional features and reduces potential biases derived from transcription inaccuracies.

### 2.3. Sentiment Analysis

Sentiment and emotion analysis was performed [20] using the NRC Word–Emotion Association Lexicon included in the SSA-DSS (Spanish Sentiment Analysis Decision Support System). This software classifies words into eight basic emotions (joy, sadness, trust, disgust, anticipation, surprise, anger, and fear) and two sentiment categories: positive and negative [4,21,22].

For each intervention, the frequency of words associated with each emotion was computed. In addition, sentiment valence was calculated as the difference between the number of positive and negative words. This approach provides a quantitative representation of sentiment valence and affective composition within doctor–patient interactions.

The NRC Emotion Lexicon was selected because it is one of the most widely used and validated lexicon-based emotion classification resources. In addition to polarity, it provides annotations for eight basic emotional categories (anger, anticipation, disgust, fear, joy, sadness, surprise, and trust), making it particularly suitable for analyzing emotional dynamics in conversational settings. Previous studies have also demonstrated its usefulness in Spanish-language healthcare contexts [5,20,22].

### 2.4. Entropy-Based Characterization of Emotional Dynamics

To characterize the distribution of emotional states, an entropy-based measure was computed from the emotional labels extracted from the text. Following previous studies on emotional dynamics in textual data [8,9,11], the relative frequency of each emotion was used to construct a probability distribution over the set of emotional categories.

Shannon entropy (*H*) was calculated as:

(1) $$H\;=\;-\;\Sigma \;pᵢ\cdot \log_{2}(pᵢ),$$

where $p_{i}$ represents the probability associated with emotion i, and n denotes the number of emotional categories considered. Probabilities were determined as the relative frequency of each emotion within an intervention, defined as the proportion of occurrences of that emotion with respect to the total number of detected emotional labels in the same intervention. No smoothing or pseudocount procedure was applied. Emotion categories with zero frequency were assigned $p_{i}=0$, with their contribution to Shannon entropy defined as $0{log}_{2}\left(0\right)=0$. For instance, one of the initial HTA interventions yields an entropy value of *H* ≈ 1.79 bits, computed from the empirical distribution of emotional labels. This reflects a situation in which several emotional categories are present with non-uniform relative frequencies, leading to a moderately dispersed emotional distribution.

To facilitate comparisons across consultations, speakers, and clinical conditions, entropy values were normalized by the maximum theoretical entropy associated with the NRC emotional framework. Since the analysis considers eight emotional categories (anger, anticipation, disgust, fear, joy, sadness, surprise, and trust), the maximum entropy is given by:

(2) $$Hₘₐₓ\;=\;\log_{2}\left(n\right)=\log_{2}(8)\;=\;3.$$

Normalized entropy was therefore computed as:

(3) $$H_{\mathrm{norm}}\;=\;\frac{H}{Hmax}$$

This normalization constrains entropy values to the interval [0, 1] and ensures that comparisons are performed on a common scale across consultations, speakers, and clinical conditions. All subsequent analyses and figures presented in this study are based on normalized entropy values.

In this study, entropy was computed at the level of individual interventions, defined as complete speaker turns. Consecutive sentences produced by the same speaker were considered part of the same intervention until the other speaker intervened. Entropy was therefore calculated over the complete text of each speaker turn, allowing for a fine-grained analysis of emotional dynamics throughout the consultation. For each actor (patient and doctor), entropy values were calculated for each intervention considering the full set of eight emotional categories. By linking the entropy values across all interventions of each speaker, the entropy curve for each consultation and speaker is constructed.

Since the number and duration of interventions varies across consultations and between speakers, a normalization procedure was applied to enable aggregation. All consultations are resampled onto a common grid consisting of 100 intervals of equal duration (defined by 101 equidistant points), representing the percentage progression of the consultation over time. This normalization facilitates the comparison of consultations with different durations and interaction structures. However, it may smooth local temporal variations and consultation-specific interaction patterns, which should be considered when interpreting aggregated trajectories. Normalization effectively rescales the sequence of interventions, allowing consultations with different durations and numbers of interventions to be represented on a common scale. The resulting curves were then averaged (point by point) to obtain a collective emotional entropy trajectory for each disease. This approach enables the examination of how emotional entropy evolves during the consultation, as well as differences between speakers (doctor vs. patient) and clinical conditions (HTA vs. DLM). Accordingly, entropy is used here strictly as an information-theoretic descriptor and not as a direct measure of psychological uncertainty, cognitive load, understanding, or decisional conflict.

To assess the robustness of the proposed measure, additional sensitivity analyses were conducted by excluding interventions containing fewer than 3, 5, and 7 words. The resulting trajectories and aggregated comparisons were subsequently compared with the original analysis.

In addition, to evaluate whether emotional entropy provides information beyond polarity, its relationship with sentiment valence was assessed through correlation analysis.

### 2.5. Analytical Framework

Following the analysis of the 30 consultations, a consistent structural pattern was identified, allowing the definition of four distinct phases in the doctor–patient interaction [1]: (i) initial emotional state, (ii) explanation of the condition, (iii) treatment and lifestyle discussion, (iv) final emotional state (see Table 1); they reflect the usual structure followed by clinicians to guide the consultation and are consistent with the Four Habits Model.

The four phases were identified by reviewing the content of each consultation rather than by applying fixed temporal boundaries. This process was carried out by the multidisciplinary research team, with particular involvement of the clinical researchers. The transitions between phases were agreed by consensus and identified separately for each consultation, so they could occur at different points in the consultation timeline. The boundaries shown in the figures correspond to the average transition points across consultations and should not be interpreted as fixed phase durations. No formal inter-rater reliability coefficient was calculated.

For each consultation, both sentiment measures (valence and emotion frequencies) and entropy values were computed across these phases. This phase-based structure allows for the examination of how emotional patterns and entropy evolve throughout the interaction.

In addition, as the entropy values were calculated at the intervention level, the analysis of the entropy evolution can be undertaken in different situations (phases, consultation, temporal windows, etc.). Moreover, the discretization of the entropy curves allows the intuitive and simple aggregate entropy information corresponding to different phases, speakers, diseases, etc. In short, it permits the identification of fluctuations in emotional entropy along several situations and contexts.

Comparative analyses were conducted for three dimensions: (i) clinical condition (HTA vs. DLM), (ii) speaker role (doctor vs. patient), (iii) consultation phase. This multi-level comparison enables the exploration of differences in emotional dynamics and entropy in contexts and roles.

By combining sentiment valence and entropy measures, this framework provides a comprehensive perspective on emotional processes in doctor–patient co-decision, capturing both the polarity of emotional expression and the structure of the distribution of emotional categories. The additional correlation analysis reinforces this interpretation. The very weak relationship observed between entropy and sentiment valence suggests that they are describing different aspects of the interaction. Valence indicates whether an intervention is predominantly positive or negative, whereas entropy reflects how varied the emotional content is. As a result, two interventions with similar valence can still differ substantially in their emotional composition. From this perspective, entropy does not replace sentiment analysis but complements it by providing additional information about the emotional dynamics of the consultation.

### 2.6. Statistical Analysis

To formally assess the main comparisons, inferential analyses were conducted at the consultation level, treating each of the 30 consultations as the independent observational unit. The 101-point normalized trajectories were used for graphical representation and were not treated as independent observations for statistical testing.

For the comparison between disease groups, each normalized entropy trajectory was summarized using the area under the curve (AUC), calculated over normalized time using the trapezoidal rule. This provided an overall measure of entropy for each consultation. AUC values were compared between HTA and DLM using a two-sided permutation test with 10,000 permutations. The mean difference between groups was accompanied by a 95% bias-corrected and accelerated (BCa) bootstrap confidence interval based on 10,000 resamples, together with Cliff’s delta as a measure of effect size.

AUC values were also calculated separately for each speaker within each consultation. Since doctor and patient values came from the same consultation, these comparisons were treated as paired. A two-sided sign-flipping permutation test with 10,000 permutations was used, together with a 95% bootstrap confidence interval for the mean paired difference and Cohen’s $d_{z}$ as a paired effect size. Disease and speaker role were also examined jointly using a linear mixed-effects model including disease, speaker role, and their interaction as fixed effects, with consultation as a random intercept.

Differences in consultation phases were examined using a second linear mixed-effects model. For this analysis, normalized mean entropy was calculated for each phase and consultation directly from the original interventions, without interpolation. Disease, consultation phase, and their interaction were included as fixed effects, with consultation again included as a random intercept. Phase-specific comparisons between HTA and DLM were obtained from estimated marginal means, and the resulting *p*-values were adjusted across the four phase comparisons using the Holm method.

Given the relatively small sample size, a sensitivity power analysis was also performed for a two-sided independent two-sample comparison with 15 consultations per group and a significance level of 0.05.

Permutation, bootstrap, effect-size, and sensitivity analyses were performed in Python (version 3.13). Linear mixed-effects models were fitted in R (version 4.6.1) using the *lme4* and *lmerTest* packages, with degrees of freedom estimated using the Satterthwaite approximation. Estimated marginal means and contrasts were obtained using the *emmeans* package.

## 3. Results

The results provide insight into the evolution of emotional entropy in doctor–patient consultations. Entropy values were analyzed at the intervention level, allowing the examination of fluctuations during the consultation, as well as differences across clinical conditions, speaker roles, and consultation phases. Overall, the results show that emotional entropy is not static but evolves dynamically throughout the interaction and exhibits distinct patterns in HTA and DLM consultations.

Unless otherwise stated, all entropy values reported in the following sections correspond to normalized entropy (Hnorm).

### 3.1. Evolution of Emotional Entropy

The horizontal axis of Figure 2 represents a temporal discretization of the consultation duration. All consultations are resampled onto a common grid consisting of 100 intervals of equal duration (defined by 101 equidistant points), representing the percentage progression of the consultation over time. The vertical axis represents the average normalized emotional entropy (H/Hmax) for each clinical condition. The results reveal dynamic fluctuations in entropy values along the interaction, indicating that emotional entropy varies throughout the consultation process.

At the beginning of the consultations, normalized emotional entropy tends to be relatively high, particularly in the HTA condition, suggesting a more heterogeneous distribution of emotional states during the initial stages of the interaction. As the consultation progresses, entropy tends to decrease, although with noticeable fluctuations, reflecting a trend towards a more concentrated distribution of the emotional categories detected.

However, stabilization is not uniform, and several local increases in entropy can be observed, indicating moments of higher normalized emotional entropy and greater diversity of emotional categories. These fluctuations are especially noticeable during intermediate stages of the consultation, which are typically associated with the discussion of diagnosis and treatment options.

Across the entire interaction, within the analyzed sample, HTA consultations tended to exhibit slightly higher and more variable normalized entropy values than DLM consultations, suggesting a more heterogeneous distribution of emotional categories. In contrast, DLM interactions tend to show lower and more stable entropy levels, indicating more concentrated emotional patterns.

The consultation-level AUC analysis showed the same pattern. Mean AUC was higher in HTA consultations (0.320 ± 0.077) than in DLM consultations (0.259 ± 0.056), with a mean difference = 0.061, 95% BCa CI [0.017, 0.110]. This difference was statistically significant in the permutation test (*p* = 0.0158; Cliff’s δ = 0.493, 95% CI [0.102, 0.804]).

Overall, these results highlight that emotional entropy evolves dynamically during consultations and differs across clinical conditions.

Figure 3 displays the individual entropy trajectories for all consultations together with the corresponding cohort mean trajectory. Although variability is evident across individual consultations, the overall temporal pattern remains broadly consistent within each clinical group. The aggregated trajectories are presented here as a descriptive summary, while the individual trajectories show the variability between consultations.

### 3.2. Doctor vs. Patient Differences

Descriptive differences in emotional entropy were observed between doctor and patient interventions for both HTA and DLM consultations.

For doctor interventions (Figure 4), normalized emotional entropy exhibits moderate variability throughout the consultation, with HTA generally showing higher and more fluctuating entropy levels than DLM. This suggests that, from the physician’s perspective, interactions in HTA contexts involve a more heterogeneous emotional profile.

In contrast, with patient interventions (Figure 5) normalized emotional entropy shows stronger fluctuations overall, with more pronounced variations during the consultation. While HTA still shows relatively higher entropy compared to DLM, patient responses exhibit sharper local variations, indicating more abrupt changes in emotional entropy.

Comparing both roles, patients tended to exhibit greater fluctuations in normalized emotional entropy than doctors, indicating greater variation in the distribution of emotional categories detected in the patient interventions. This effect is particularly noticeable in intermediate and later stages of the consultation, where patient entropy shows more irregular patterns.

Although the trajectories showed descriptive differences between doctors and patients, the mixed-effects analysis did not show a significant overall effect of speaker role (F(1,28) = 1.02, *p* = 0.322) or a significant disease-by-role interaction (F(1,28) = 2.26, *p* = 0.144). Paired AUC comparisons also showed no significant doctor–patient differences within HTA (mean difference = −0.007, 95% CI [−0.047, 0.032], *p* = 0.739) or DLM (mean difference = 0.036, 95% CI [−0.0002, 0.071], *p* = 0.088). However, the mixed-effects model showed a significant overall effect of clinical condition (F(1,28) = 6.19, *p* = 0.019), consistent with the higher entropy observed in HTA consultations.

### 3.3. Emotional Entropy Across Consultation Phases

To further explore how normalized emotional entropy evolves during the consultation, entropy values were aggregated for the four phases described in Section 2.5. Mean normalized emotional entropy values were calculated for each phase by aggregating all interventions belonging to that phase.

As shown in Figure 6, the evolution of entropy across phases was not identical in the two conditions. In HTA consultations, mean entropy decreased from Phase 1 (0.443) to Phase 2 (0.395) and Phase 3 (0.301), before increasing again in Phase 4 (0.367). In DLM consultations, entropy increased slightly from Phase 1 (0.369) to Phase 2 (0.408), then decreased in Phase 3 (0.241) and remained relatively stable in the final phase (0.251). Points represent mean entropy values and error bars indicate standard deviation. For visual clarity, error bars are slightly horizontally shifted and do not represent differences in phase position.

In Phase 3, corresponding to the discussion of treatment and lifestyle alternatives, normalized emotional entropy decreases for both HTA and DLM. This indicates a shift towards a more concentrated distribution of detected emotional categories during this stage of the consultation.

The mixed-effects analysis confirmed that entropy varied across consultation phases (F(3,84) = 8.57, *p* < 0.001) and also showed an overall difference between clinical conditions (F(1,28) = 4.62, *p* = 0.040). However, the disease-by-phase interaction was not significant (F(3,84) = 1.36, *p* = 0.260). When HTA and DLM were compared within each phase, none of the differences remained statistically significant after Holm correction (adjusted *p* = 0.397, 0.779, 0.438, and 0.074 for Phases 1–4, respectively).

Overall, the results indicate that normalized emotional entropy is not constant throughout the consultation but varies in a structured manner during the consultation.

### 3.4. Robustness Analysis

To evaluate the robustness of the entropy estimates, we conducted a series of sensitivity analyses excluding interventions containing fewer than 3, 5, and 7 words. The overall patterns observed in the original dataset remained remarkably stable across all filtering thresholds. In particular, average normalized entropy consistently remained higher in HTA consultations than in DLM consultations (HTA: 0.297, 0.330, 0.353, and 0.384 vs. DLM: 0.249, 0.280, 0.313, and 0.343 for the original dataset and the <3, <5, and <7-word thresholds, respectively). Likewise, the distinction between doctor and patient trajectories and the general temporal evolution of emotional entropy were preserved. These findings suggest that the reported patterns are not driven by isolated very short utterances.

Because the main analyses rely on average trajectories across consultations, we also assessed the consistency of the individual consultations within each clinical group. No statistically significant differences were observed among consultations within the HTA cohort (Kruskal–Wallis H = 21.68, *p* = 0.0855) or within the DLM cohort (H = 7.70, *p* = 0.9041). These findings indicate that no statistically significant differences were detected among consultations within either cohort. Aggregated trajectories are presented as descriptive summaries of group-level emotional dynamics, while individual variability is shown in Figure 3.

We also explored whether entropy values were simply reflecting intervention length. Correlation analyses showed a moderate positive association between the number of words and entropy (Pearson r = 0.547; Spearman ρ = 0.627), indicating that longer interventions tend to contain a greater diversity of emotional categories. However, the stability of the results after removing short interventions suggests that the main patterns are not entirely driven by the shortest interventions, although intervention length may still contribute to the observed entropy values.

We also examined the relationship between emotional entropy and sentiment valence. The correlation between both measures was negligible (Pearson r = −0.077), suggesting that they reflect different aspects of emotional communication. While sentiment valence indicates whether an intervention is predominantly positive or negative, entropy reflects how many different emotional categories are expressed and how they are distributed. In several interventions, sentiment valence remained close to neutral whereas entropy values were relatively high, indicating the simultaneous presence of multiple emotions. These results suggest that entropy captures aspects of emotional expression that are not reflected by sentiment polarity alone and therefore offers a useful complementary perspective on emotional dynamics during the consultation.

Finally, we considered the implications of the sample size for the between-group comparisons. With 15 consultations per group and a two-sided α = 0.05, an effect size of approximately d = 1.06 would be required to achieve 80% power. This suggests that the between-group analysis was better suited to detecting large differences, while smaller effects may not have been reliably detected.

## 4. Discussion

This study set out to examine emotional dynamics in doctor–patient interactions from a different perspective, focusing not only on the type or polarity of emotions, but also on how emotional categories are distributed within the interaction. By using normalized entropy as a measure of emotional diversity and distributional structure, the analysis provides an additional layer of information that complements traditional sentiment-based approaches [4]. Entropy-based metrics have been increasingly used to characterize complexity and information structure in human and information systems, including applications to emotional and behavioral data [9,11].

The patterns observed in this study are broadly consistent with previous research on healthcare communication, shared decision-making, and sentiment-based analyses of clinical interactions. While previous studies have highlighted the importance of emotional processes during information exchange and treatment discussions [23,24,25,26], the present work extends these approaches by examining the distribution and diversity of emotional categories through an entropy-based perspective.

Before discussing the results, it is important to clarify the interpretation of emotional entropy in the present study. The proposed measure is computed from the distribution of detected emotional categories and therefore captures the degree of concentration or dispersion of detected emotional categories within an interaction. In this sense, entropy is interpreted as an information-theoretic property of the emotional distribution. Although emotional entropy may be associated with uncertainty in some contexts, it should not be considered a direct measure of psychological uncertainty, confusion, or decisional conflict, which were not explicitly assessed in this study.

Additional robustness analyses further support this interpretation. The main findings remained stable after excluding very short interventions, and moderate correlations were observed between intervention length and entropy. Furthermore, entropy showed a negligible association with sentiment valence, suggesting that it captures a distinct structural dimension of emotional expression rather than merely reflecting emotional polarity.

Accordingly, the present results should be interpreted as evidence that emotional entropy varies systematically across consultation contexts and phases, rather than as evidence of a specific psychological construct. While these patterns indicate systematic differences in the distribution of emotional categories, establishing construct validity more directly would require future studies incorporating independent measures such as perceived uncertainty, decisional conflict, anxiety, or patient understanding.

The results show that emotional entropy is not constant throughout the consultation. At the intervention level, the descriptive trajectories suggested greater local fluctuations in patient contributions than in doctor contributions. However, the inferential analyses did not show a significant overall effect of speaker role, indicating that these descriptive differences should be interpreted with caution. Previous studies have highlighted the relevance of emotional processes and uncertainty in clinical encounters and shared decision-making [23,24,25]. However, these psychological processes were not directly assessed in the present study and therefore cannot be inferred from the observed entropy patterns.

Differences between clinical conditions were also observed. Although HTA and DLM share similar characteristics as cardiovascular risk factors, HTA consultations showed higher overall entropy than DLM consultations, a difference that was also supported by the consultation-level AUC analysis (mean difference = 0.061, *p* = 0.0158). These differences may be related to the distinct clinical characteristics of the two conditions, including differences in symptom perception and perceived risk [27,28]. Previous research has shown that patients’ perception of risk and symptom visibility can significantly influence their emotional responses and engagement in clinical decision-making, with more salient or symptomatic conditions often associated with greater emotional variability [29]. In line with previous findings based on sentiment valence, where emotional differences between conditions were also observed [1], the present results suggest that such differences are not only reflected in the polarity of emotions but also in the structure of their emotional distributions. This finding complements previous sentiment-based analyses by showing that emotional distinctions between clinical conditions can also be characterized in terms of the diversity and concentration of emotional categories.

The phase-based analysis provides further insight into how emotional entropy evolves in the course of the consultation. Emotional entropy varied in consultation phases; this was also supported by the mixed-effects analysis (*p* < 0.001). Although the trajectories were not identical in HTA and DLM consultations, the disease-by-phase interaction was not statistically significant (*p* = 0.260). In particular, the explanation phase coincides with the introduction and discussion of new clinical information [30]. This may help contextualize the distribution of emotional categories observed during this phase, although entropy itself does not directly measure information processing. In contrast, the treatment phase is associated with a reduction in entropy, which may reflect a more concentrated distribution of emotional categories during this stage of the interaction [25,26]. Towards the end of the consultation, entropy increased again in HTA and remained relatively stable in DLM. These descriptive patterns may reflect differences in the distribution of emotional categories during the final stage of the interaction, although the phase-specific comparisons between clinical conditions were not statistically significant after correction for multiple comparisons.

These findings are consistent with previous results obtained using sentiment valence in similar settings, where emotional changes were more pronounced in the explanation phase and more stable during treatment discussions [1]. Taken together, both perspectives provide complementary information on how emotional patterns evolve across the different stages of the consultation. In this sense, entropy does not replace sentiment analysis but complements it by capturing the diversity and distributional structure of emotional states throughout the interaction.

From a practical perspective, incorporating entropy into the analysis of clinical interactions may contribute to a better understanding of co-decision processes. Phases with higher entropy may indicate moments where a broader range of emotional categories is expressed, potentially signaling more complex emotional dynamics [30]. Conversely, lower entropy may reflect more stable emotional patterns during this stage of the consultation, which are key elements of effective shared decision making [26].

From a methodological standpoint, this study highlights the value of combining sentiment analysis with information-theoretic measures to better capture emotional dynamics in clinical interactions. While sentiment analysis provides information about the polarity and type of emotions expressed, entropy offers insight into the distribution and diversity of emotional categories, allowing for a more nuanced understanding of emotional processes. Entropy does not replace traditional sentiment metrics but complements them by capturing the structure and diversity of emotional distributions [9,11]. This combined approach enables a more comprehensive representation of doctor–patient interactions, where both the direction and the structure of emotional responses are relevant for understanding co-decision processes. More broadly, these findings support the use of entropy-based measures as a useful tool for analyzing emotional complexity in decision-making contexts, supporting the relevance of entropy as an information-theoretic measure to characterize the distributional structure of emotional states in human interactions.

These results suggest that emotional entropy captures a structural dimension of doctor–patient interactions that cannot be fully described by sentiment polarity alone.

The contribution of this study does not lie in the introduction of a novel entropy measure, but in the application of emotional entropy as a complementary analytical perspective in doctor–patient interactions. While sentiment analysis provides information about emotional polarity, entropy offers information about the distribution and diversity of emotional categories. Together, these measures provide a richer description of emotional dynamics than either approach alone.

Previous healthcare-related studies have also demonstrated the usefulness of entropy-based measures for characterizing affective and behavioral phenomena. For example, entropy has been successfully applied to the analysis of laughter patterns associated with depression, providing information about the complexity and structure of emotional expression [31]. In this context, the present study extends the application of entropy-based approaches to the analysis of emotional distributions in doctor–patient communication.

This study has several limitations that should be considered when interpreting the results. First, the analysis is based on a relatively small sample of 30 consultations from a specific primary care setting, which limits the statistical power of the analyses and the generalizability of the findings. The sensitivity analysis indicated that the between-group comparison was primarily suited to detecting large effects so smaller differences may not have been identified. In addition, demographic information such as patient age and sex was not available due to the anonymization procedures applied to the dataset. Consequently, it was not possible to assess whether emotional entropy patterns differed across demographic subgroups. Future studies incorporating demographic metadata could help determine whether emotional entropy patterns vary across different patient populations. Second, the study focuses on two specific clinical conditions, hypertension (HTA) and dyslipidemia (DLM), and the results may not be directly transferable to other contexts.

An additional methodological consideration relates to the linguistic context of the study. The analysis was conducted on Spanish-language consultations using a Spanish version of the NRC Emotion Lexicon. Although NRC-based emotion analysis has been successfully applied in several healthcare-related studies [5,20,22], the identification of emotions depends on predefined lexical resources and may not fully capture contextual phenomena such as negation, contextual dependencies, cultural factors, or domain-specific nuances of emotional expression. Future research could explore domain-adapted lexicons and contextual language models to complement emotion analysis in clinical communication settings. Although sensitivity analyses demonstrated the stability of the results after excluding short interventions, entropy estimates may still be influenced by sparse emotional counts in individual interventions. Future studies could explore alternative smoothing procedures and larger datasets to further evaluate the robustness of entropy-based emotional indicators. In addition, the moderate association observed between intervention length and entropy suggests that text length may remain a potential source of variability. Although the main patterns remained stable across the sensitivity analyses, future studies should further investigate normalization and modelling strategies that explicitly account for length-related effects.

Finally, while entropy provides a robust and well-established information-theoretic measure to quantify the distribution and diversity of emotional states, its application in this study is based on discrete emotion categories and does not explicitly incorporate aspects such as emotional intensity or contextual semantics. Future research could extend this approach to larger datasets and explore enriched formulations of entropy that integrate additional dimensions of emotional complexity. Entropy can therefore be interpreted as a structural indicator of emotional distributional patterns in human decision-making processes, extending its application to the analysis of clinical interactions.

## 5. Conclusions

This study examined emotional dynamics in doctor–patient interactions by integrating sentiment analysis with entropy-based measures. By using normalized entropy as an indicator of emotional diversity and distributional structure, the analysis provides a complementary perspective to sentiment analysis by capturing the diversity and distribution of detected emotional categories.

The results show that emotional entropy varies throughout the consultation and across consultation phases. Overall entropy was higher in HTA than in DLM consultations, a difference supported by the consultation-level analysis. Although the descriptive trajectories suggested differences between doctors and patients, no significant overall effect of speaker role was found. Entropy also varied significantly in consultation phases, while the disease-by-phase interaction was not significant.

Additional robustness analyses confirmed that the observed patterns remained stable after excluding very short interventions and across alternative filtering thresholds. Furthermore, the negligible association between normalized emotional entropy and sentiment valence suggests that both measures capture complementary dimensions of emotional communication. Together, these findings seem to show that normalized emotional entropy captures differences in how emotion categories are distributed across interventions. However, the observed association between entropy and intervention length indicates that longer interventions may partly contribute to these differences by providing more emotional information. This should be considered when interpreting the results.

From an applied perspective, these findings highlight the potential of entropy-based approaches to improve the understanding of co-decision processes in clinical settings. By identifying moments of increased emotional entropy, such methods may provide additional quantitative information for the analysis of doctor–patient communication. However, these findings should be interpreted in the light of the limited sample size and the specific clinical context analyzed. Future research could extend this approach to larger datasets and additional medical conditions, as well as explore its integration into real-time decision support systems.

The study illustrates how normalized emotional entropy can be used to quantitatively describe emotional patterns and their evolution during clinical consultations.

Emotional entropy should therefore be interpreted as a structural descriptor of the distribution of emotional categories rather than as a direct indicator of psychological uncertainty, understanding, or decisional conflict. Future work combining entropy measures with independent psychological assessments will be necessary to further evaluate its construct validity in healthcare settings.

## Figures and Tables

**Figure 1:**
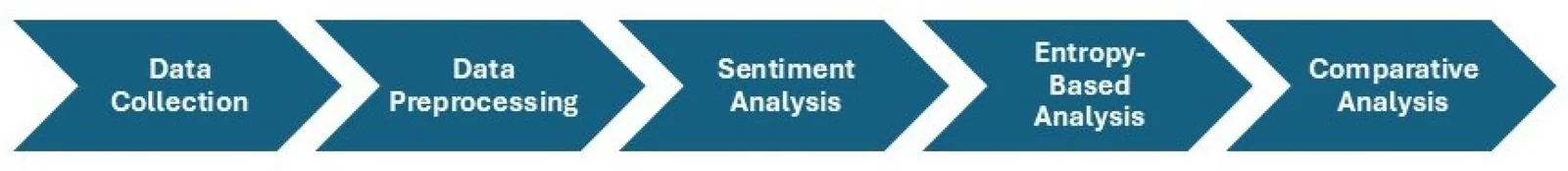
Pipeline for sentiment and entropy-based analysis of doctor–patient consultations.

**Figure 2:**
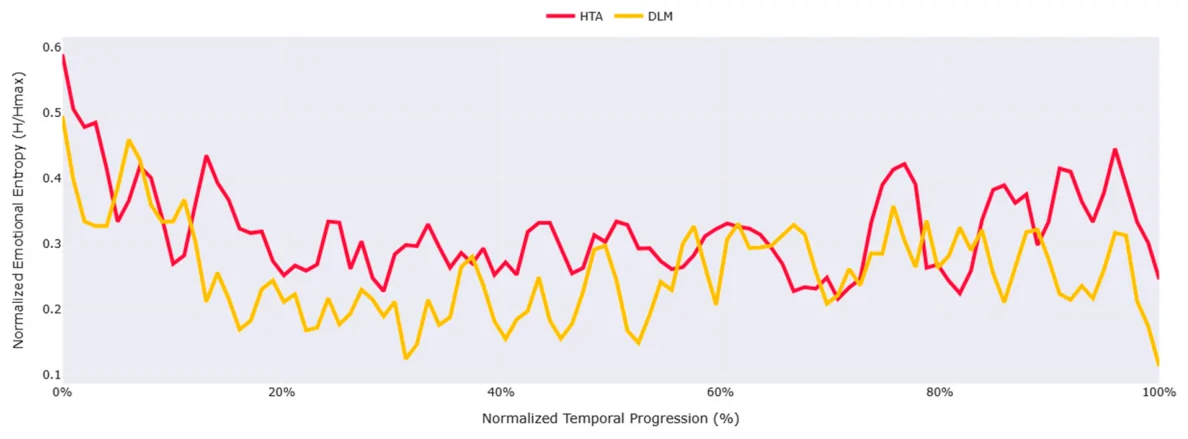
Evolution of normalized emotional entropy across consultations for HTA and DLM.

**Figure 3:**
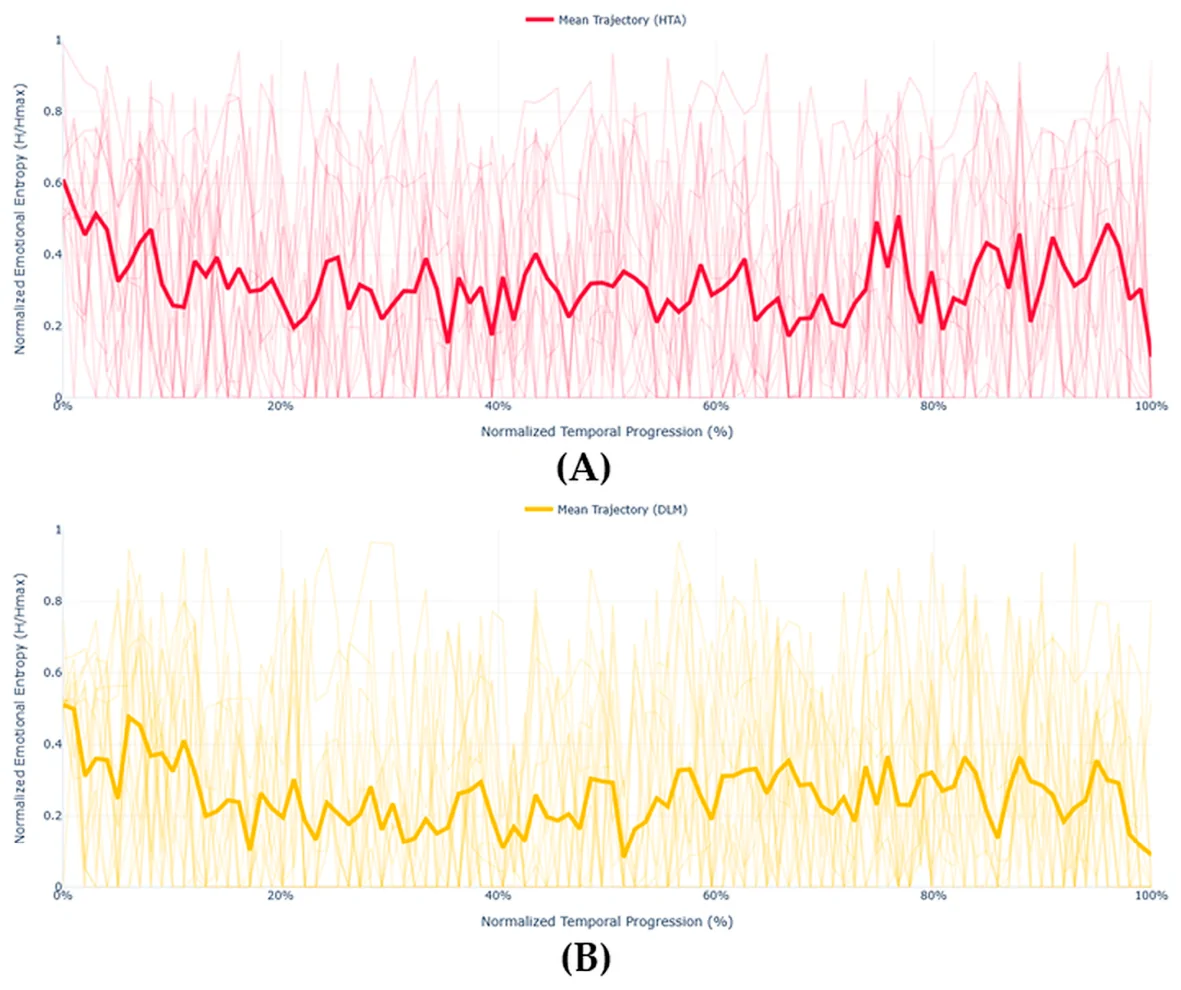
Individual and mean normalized emotional entropy trajectories for the consultations. Light lines represent individual consultation trajectories, while the darker solid line represents the mean trajectory. (**A**) HTA consultations (*n* = 15). (**B**) DLM consultations (*n* = 15).

**Figure 4:**
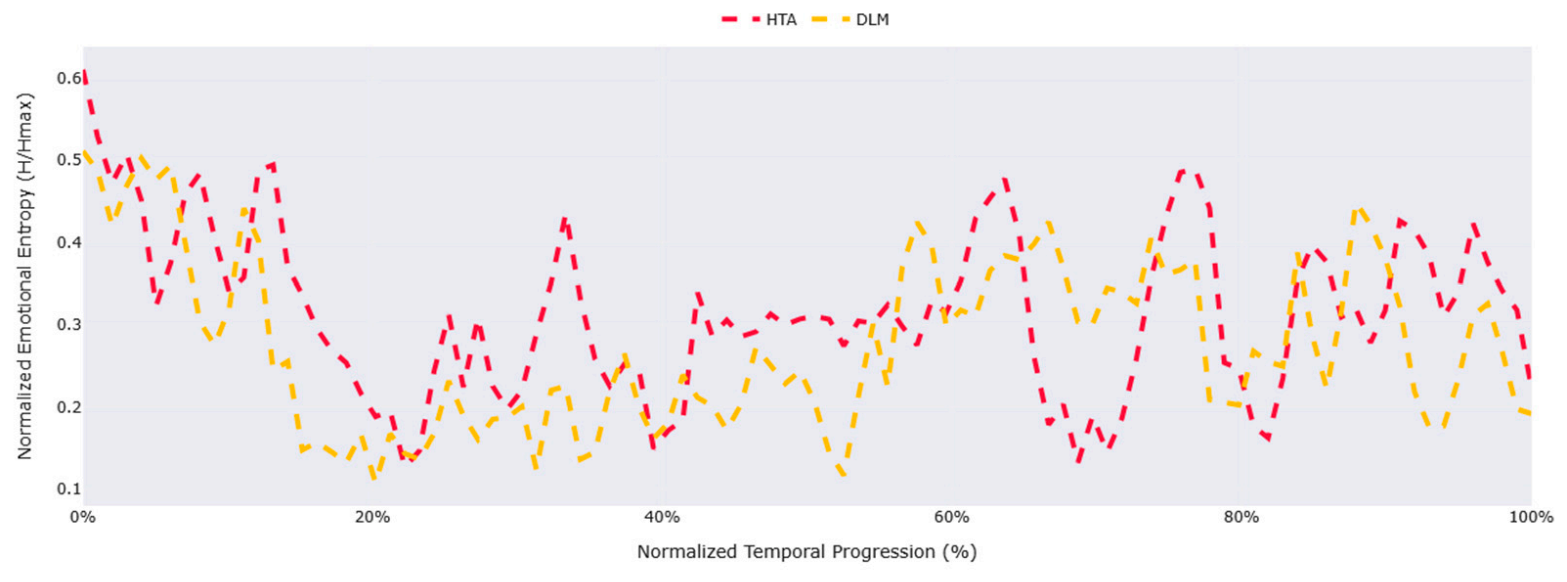
Evolution of normalized emotional entropy for doctor interventions in HTA and DLM.

**Figure 5:**
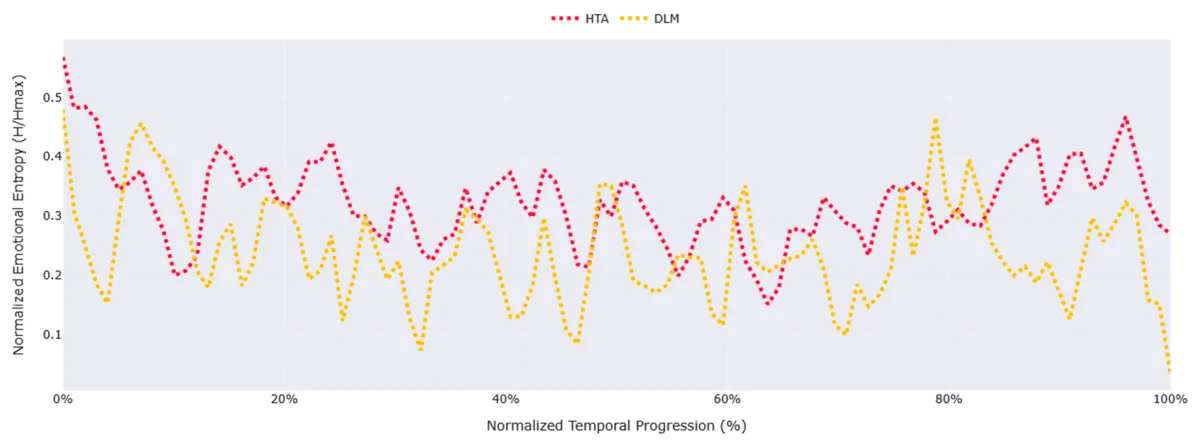
Evolution of normalized emotional entropy for patient interventions in HTA and DLM.

**Figure 6:**
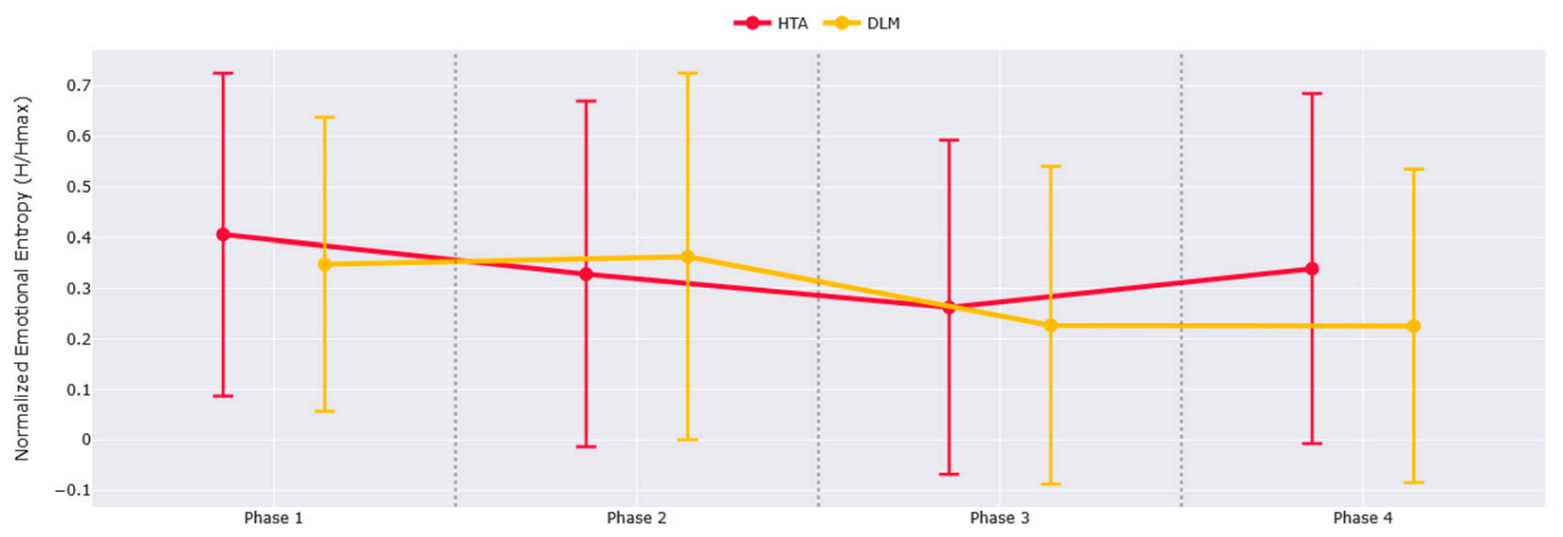
Mean normalized emotional entropy across consultation phases in HTA and DLM consultations. Vertical dashed lines indicate the boundaries between consultation phases.

**Table 1:** Four-phase structure of the doctor–patient consultation.

Phase	Description
Phase 1. Initial emotional state	Initial assessment of emotions and building trust through balanced interaction
Phase 2. Explaining the condition and medical history to the patient	Explanation of the diagnosis and review of the medical history, with a discussion led by the doctor to clarify the conditions and enable the patient to make informed decisions
Phase 3. Treatment and lifestyle alternatives	Lifestyle modification, encouraging patient-led discussion about habits and challenges to promote treatment adherence and self-management autonomy
Phase 4. Final emotional state	Completion of the assessment of emotional progress and commitment to treatment through balanced dialogue between doctor and patient

## Data Availability

The data supporting the findings of this study are not publicly available due to privacy and ethical restrictions, as they involve confidential clinical data from doctor–patient consultations.

## Abbreviations

The following abbreviations are used in this manuscript:

CEICA	Ethics Committee of Aragón
DLM	Dyslipidemia
HC-DSS	Healthcare Cognitive Decision Support System
HTA	Hypertension
NLP	Natural Language Processing
NRC	NRC Word–Emotion Association Lexicon
SDM	Shared Decision Making
SSA-DSS	Spanish Sentiment Analysis Decision Support System
